# Supplementary material for: Lack of the Ig cell adhesion molecule BT-IgSF (IgSF11) induced behavioral changes in the open maze, water maze and resident intruder test
Source: PLoS One. 2023 Jan 6;18(1):e0280133. doi: 10.1371/journal.pone.0280133 (PMC9821459; doi:10.1371/journal.pone.0280133)
Supplement: Attachment — Submitted filename: Response to Reviewers PONE-D-22-21240.docx [file pone.0280133.s005.docx]

We thank for the overall positive evaluation of our manuscript.

Referee 1 asked for additional experiments/information on

1) the AMPA receptor (electrophysiology or immunohistochemistry) and

2) on gap junctional communication between astrocytes (immunohistochemistry)

in the absence of BT-IgSF

3) whether this mutation impacts sociability

We agree with referee 1 that such data strongly support our interpretations of the behavioral deficits in the absence of BT-IgSF which are drawn in our manuscript. Indeed, such data on the AMPA receptor including electrophysiological measurements and immunohistochemistry in the absence of BT-IgSF (also termed IgSF11) have already been published by Jang et al. 2015 (ref. #7). Furthermore, studies on gap junctional communication between astrocytes including immunohistochemistry, biochemistry and biocytin spreading in the cortex and hippocampus in the absence of BT-IgSF have been recently published by Pelz et al., 2022 (ref. #14). Therefore, now we have cited and discussed these published data in detail in the conclusion paragraph of the revised version of our manuscript. We feel that these additions improved our manuscript in that they illustrate the contribution of BT-IgSF to network functions in the nervous system. The increased aggression of *BT-IgSF11^-/-^* males reported in our manuscript dominates sociability with littermates and their infertility affects interaction with females.

All changes that we added are marked in red in our manuscript file "Revised Manuscript with Track Changes PONE-D-22-21240". All original data are now provided in supplementary files.

We hope very much that with these additions our manuscript is now acceptable for publication in PLOS ONE.
